# Whole‐genome analysis of influenza A(H1N1)pdm09 viruses isolated in Uganda from 2009 to 2011

**DOI:** 10.1111/irv.12401

**Published:** 2016-07-19

**Authors:** Denis K. Byarugaba, Bernard Erima, Monica Millard, Hannah Kibuuka, Luswa Lkwago, Josephine Bwogi, Derrick Mimbe, Jocelyn B. Kiconco, Titus Tugume, Edison A. Mworozi, Jasmine Turner, Pamela P. Mckenzie, Richard R. J. Webby, Robert G. Webster, Charlotte Foret, Mariette F. Ducatez, Rodney Coldren, Fred Wabwire‐Mangen, Scott Krauss

**Affiliations:** ^1^ College of Veterinary MedicineMakerere UniversityKampalaUganda; ^2^ Makerere University Walter Reed ProjectKampalaUganda; ^3^ Ministry of HealthKampalaUganda; ^4^ Uganda Virus Research InstituteEntebbeUganda; ^5^ College of Health SciencesMakerere UniversityKampalaUganda; ^6^ Department of Infectious DiseasesSt. Jude Children's Research HospitalMemphisTNUSA; ^7^ IHAPINRAENVTUniversité de ToulouseToulouseFrance; ^8^ U.S. Army Medical Research Directorate‐KenyaU.S. EmbassyNairobiKenya

**Keywords:** influenza A(H1N1)pdm09, Uganda, whole‐genome sequencing

## Abstract

We report a whole‐genome analysis of 19 influenza A(H1N1)pdm09 isolates from four Ugandan hospitals between 2009 and 2011. The isolates differed from the vaccine strain A/California/07/2009 by three amino acid substitutions P100S, S220T, and I338V in the hemagglutinin and by two amino acid substitutions V106I and N248D in the neuraminidase proteins with consistent mutations in all gene segments distinguishing isolates from the 2009/2010 to 2010/2011 seasons. Phylogenetic analysis showed low genetic evolution, with genetic distances of 0%–1.3% and 0.1%–1.6% for HA and NA genes, respectively. The amino acid substitutions did not lead to antigenic differences from the reference strains.

## 1 Introduction

Despite increased capabilities for the detection and surveillance of influenza viruses in many parts of the world,^1^ whole‐genomic data on the viruses from sub‐Saharan Africa remain limited. Yet whole‐genome sequencing provides the needed evidence for scientifically sound public health interventions including vaccine development. Due to constant viral antigenic evolution, it is important to continually examine the evolutionary changes in the strains for appropriate vaccine selection each year.^2^ The influenza A(H1N1)pdm09 virus was first reported in Uganda in July 2009 and subsequently spread and established rapidly within the population where it was thereafter routinely detected. In this study, we report the whole‐genome analysis of and genetic variations among the pandemic influenza A(H1N1)pdm09 viruses isolated between July 2009 and May 2011 from four hospital sites in Uganda.

## 2 Materials and Methods

The study was conducted in two influenza seasons: the 2009/2010 season from July 2009 to February 2010 and the 2010/2011 season from July 2010 to April 2011. Samples from patients were collected from four Ugandan hospital‐based sentinel surveillance sites: Mulago National Referral Hospital in Kampala (central Uganda), Jinja Regional Referral Hospital in Jinja District (eastern Uganda), Bugiri District Hospital in Bugiri District (eastern Uganda), and Gulu Regional Referral Hospital in Gulu District (northern Uganda). Only individuals who had a fever (temperature ≥38°C) plus either a cough or a sore throat in the last 72 hour of reporting to the outpatient clinic were enrolled in the study. Samples were collected and screened for pandemic influenza using the Centers for Disease Control and Prevention (CDC) protocol for real‐time reverse transcription (RT‐PCR) for influenza A (H1N1).^3^ The viruses were isolated in MDCK cells. Three representative isolates were compared against the reference strains in a hemagglutination inhibition (HI) test^4^ using 0.5% turkey erythrocytes for antigenic differences. For whole‐genome sequencing, RNA was extracted from isolates with the RNeasy^®^ mini kit (Qiagen) as per the manufacturer's protocol. All the eight segments were amplified in a single reaction by RT‐PCR using Uni‐12, Uni‐13, and polymerase gene primers as previously described^5^ and SuperScript III One‐Step RT‐PCR System with Platinum^®^ *Taq* High Fidelity DNA Polymerase (Invitrogen). PCR products were gel‐extracted and purified using the Illustra GFX^™^ PCR DNA and Gel Band Purification Kit (GE Healthcare, Pittsburgh, Pennsylvania, USA) per the manufacturer's instructions. PCR products were sequenced by the Sanger sequencing method at the National Institute of Allergy and Infectious Diseases Center of Excellence in Influenza Research and Surveillance/WHO Collaborating Center for the Ecology of Influenza in Animals at St. Jude Children's Research Hospital (St. Jude), Memphis, TN. Nucleotide sequences obtained were aligned by ClustalW and edited with Bioedit Software version 5.0.9.^6^ Phylogenetic analysis of the Ugandan, the pandemic H1N1 vaccine strain (A/California/07/2009), and other related sequences from the GenBank was performed. Sequence feature (SF) variant type analysis was performed from the Influenza Research Database as described previously^7^ to predict whether the isolates carried specific SFs of importance. The sequences were deposited into GenBank with accession numbers KJ690389–KJ690546.

## 3 Ethical approval

The study was approved by Makerere University School of Public Health Institutional Review Board (MUSPH #020), the US Army Research and Material Command (MRMC HSRRB/HRPO #RV231/A‐1427.1), and the Uganda National Council for Science and Technology (UNCST #HS377).

## 4 Results

**Figure 1:**
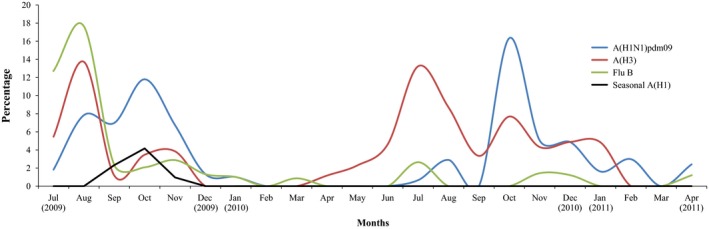
Occurrence and seasonality of the influenza A(H1N1)pdm09 isolates during the study period (July 2009 to April 2011)

**Figure 2:**
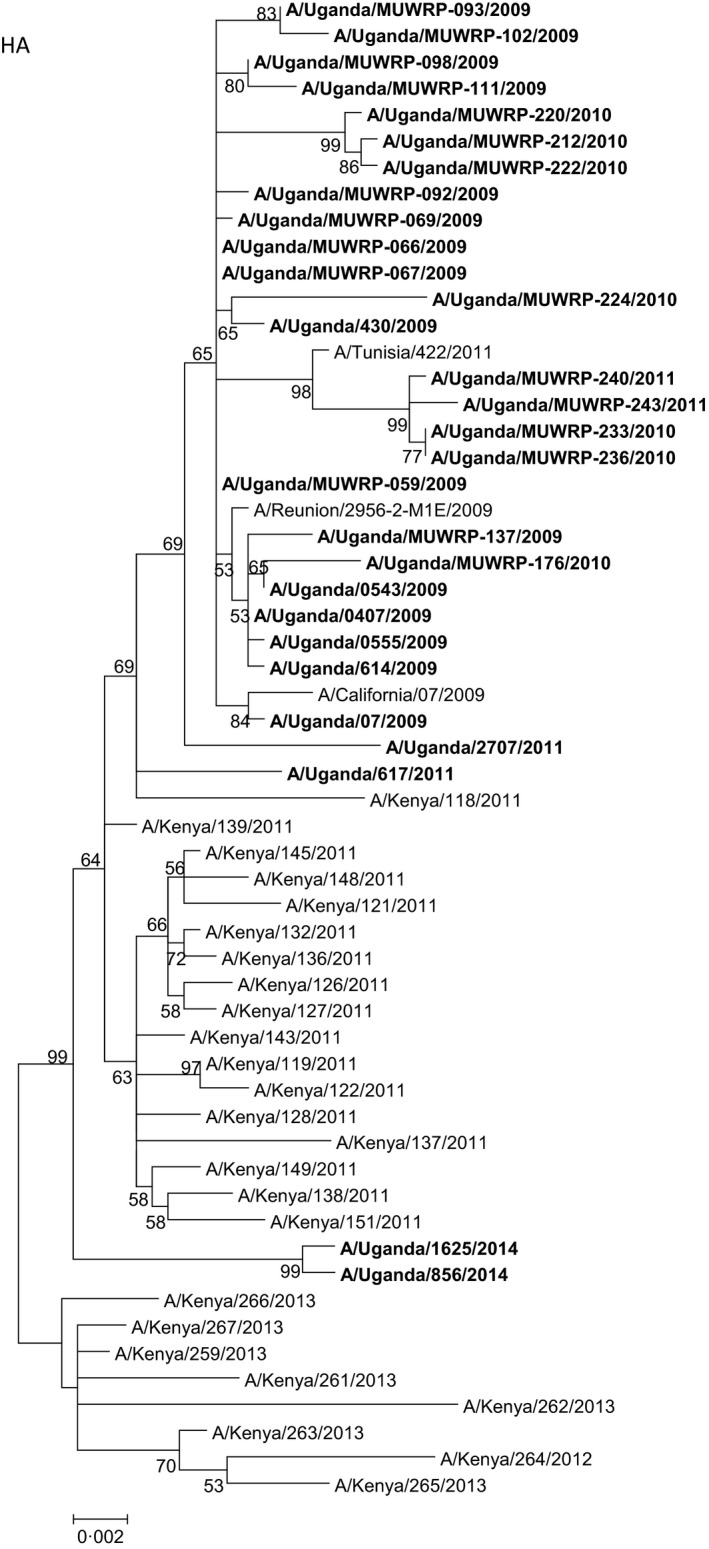
Phylogenetic tree of the hemagglutinin (HA) gene segment of the Ugandan influenza A(H1N1)pdm09 viruses (in bold font) at the nucleotide level. The HA sequences of Ugandan H1N1 isolates were compared with relevant virus sequences available in GenBank: sequences from representative influenza A(H1N1)pdm09 isolates from Africa and closest blast hits from different regions of the world. Ugandan sequences available in the GISAID Epiflu database were also used (GISAID isolates ids: EPI_ISL_94767, EPI_ISL_105881, EPI_ISL_176803, EPI_ISL_176806). Sequences of vaccine strains [A/California/07/2009 (H1N1pdm09) and A/Brisbane/59/2007 (seasonal H1N1)] were also included. Bootstrap values (500 replicates) >50 are indicated on the nodes.*indicates partial sequence data

Influenza A(H1N1)pdm09 was the second most predominant strain during the period (36%; 73/199 influenza isolates) which by December 2009 had completely replaced the seasonal influenza H1N1 (Fig 1). Virus isolates did not show significant antigenic differences from the vaccine strain A/California/07/2009 (Table 1) in antigenic tests. Whole‐genome analysis of 19 influenza A(H1N1)pdm09 isolates showed that they differed from A/California/07/2009 by three amino acid substitutions P100S, S220T, and I338V in the HA segment and two amino acid substitutions V106I and N248D in the neuraminidase (NA) segment. The genetic variation among the isolates recovered was low, with genetic distances of 0%–1.3% for HA (Fig. 2). The influenza A(H1N1)pdm09 strains isolated in July and August 2009 closely matched the A/California/07/2009 vaccine strain (except for the few amino acid differences detected in all Uganda isolates), but those isolated subsequently had additional differences from the A/California/07/2009 strain (Table 2). While there were no age‐ or gender‐related differences in the isolates, it was noted that the strains isolated from Jinja Hospital could be differentiated from the strains from the other hospital sites by an amino acid Y366F substitution in the HA gene. Most of the 2010 influenza A(H1N1)pdm09 strains had the amino acid substitutions V36I, S145P, I303V, and I341V. The late 2010 and the 2011 influenza A(H1N1)pdm09 strains were further defined by amino acid substitutions A151, S200P, and E516K, and there were additional amino acid substitutions in the 2011 isolates as shown in Table 2.

**Table 1:** Antigenic comparison of representative Ugandan influenza A(H1N1)pdm09 virus isolates with reference strains

Virus strain	HI titers	
	Reference antisera	
Reference strain	F.2009‐4 α A/California/4/2009	F.2009‐8 α A/Tennessee 1‐560/2009
A/Tennessee/1‐560/2009 (H1N1)	160	160
A/California/04/2009 (H1N1)	80	160
Test strain		
A/Uganda/MUWRP‐098/2009 (H1N1)	320	160
A/Uganda/MUWRP‐220/2010 (H1N1)	640	320
A/Uganda/MUWRP‐233/2010 (H1N1)	320	320

HI, hemagglutination inhibition.

**Table 2:** Ugandan influenza A(H1N1)pdm09 isolates (n = 19) included in the study and their genetic substitutions in the HA and NA proteins compared with the A/California/07/2009 vaccine strain

Virus strain	Date of sample collection	Patient age (months)	Patient gender	Hospital	Amino acid substitutions in the HA compared to A/California/07/2009	Amino acid substitutions in the NA compared to A/California/07/2009
A/Uganda/MUWRP‐059/2009(H1N1)	July 22, 2009	39	F	Mulago	P100S, S220T, I338V	V106I, N248D
A/Uganda/MUWRP‐092/2009(H1N1)	Sep 21, 2009	84	M	Mulago	P100S, S220T, I338V	
A/Uganda/MUWRP‐066/2009(H1N1)	Aug 5, 2009	36	M	Mulago	P100S, S220T, I338V	V106I, N248D, N309D
A/Uganda/MUWRP‐067/2009(H1N1)	Aug 5, 2009	67	F	Mulago	P100S, S220T, I338V	V106I, N248D, N309D
A/Uganda/MUWRP‐069/2009(H1N1)	Aug 7, 2009	56	F	Mulago	P100S, S220T, I338V, H45Q	V106I, N248D, N309D
A/Uganda/MUWRP‐111/2009(H1N1)	Oct 19, 2009	12	F	Jinja	P100S, S220T, I338V, Y366F	V106I, N248D
A/Uganda/MUWRP‐098/2009(H1N1)	Sep 28, 2009	168	M	Jinja	P100S, S220T, I338V, Y366F	V106I, N248D, I393V, G454S
A/Uganda/MUWRP‐102/2009(H1N1)	Oct 5, 2009	240	F	Jinja	P100S, S220T, I338V, V64I, A65S, N146R, G187, Y366F, H370L	V106I, N248D
A/Uganda/MUWRP‐093/2009(H1N1)	Sep 23, 2009	15	F	Mulago	P100S, S220T, I338V, V64I, A65S, N146R, G187	V106I, N248D
A/Uganda/MUWRP‐137/2009(H1N1)	Nov 3, 2009	42	M	Jinja	P100S, S220T, I338V, D239E	V106I, N248D, M15V, I17V, I188T
A/Uganda/MUWRP‐176/2010(H1N1)	July 6, 2010	29	F	Mulago	P100S, S220T, I338V, I133M, H155, D239E	V106I, N248D, M15V, I17V, I188T
A/Uganda/MUWRP‐212/2010(H1N1)	Oct 22, 2010	84	M	Gulu	P100S, S220T, I338V, V36I, S145P, I303V, 1341V	V106I, N248D
A/Uganda/MUWRP‐220/2010(H1N1)	Oct 26, 2010	6	M	Gulu	P100S, S220T, I338V, V36I, S145P, I303V, I341V	V106I, N248D
A/Uganda/MUWRP‐222/2010(H1N1)	Oct 28, 2010	24	M	Gulu	P100S, S220T, I338V, V36I, S145P, I303V, I341V	V106I, N248D
A/Uganda/MUWRP‐224/2010(H1N1)	Nov 11, 2010	7	F	Jinja	P100S, S220T, I338V, V36I, S145P, I303V, I341V	Q39R, S70N; N190S, Q309S, N386S
A/Uganda/MUWRP‐233/2010(H1N1)	Dec 9, 2010	18	M	Mulago	P100S, S220T, I338V, A151, S200P, E516K	R220K, Q313R, I389K, V394I
A/Uganda/MUWRP‐236/2010(H1N1)	Dec 17, 2010	22	M	Mulago	P100S, S220T, I338V, A151, S200P, E516K	Q78R, R220K, Q313R, I389K, V394I
A/Uganda/MUWRP‐240/2011(H1N1)	Feb 7, 2011	18	F	Mulago	A151, S200P, E516K, S207G	N44I, R220K, Q313R, I389K, V394I
A/Uganda/MUWRP‐243/2011(H1N1)	Apr 11, 2011	228	F	Jinja	A151, S200P, E516K, E252G, K475E	R220K, Q313R, I389K, V394I

HA, hemagglutinin; NA, neuraminidase.

**Figure 3:**
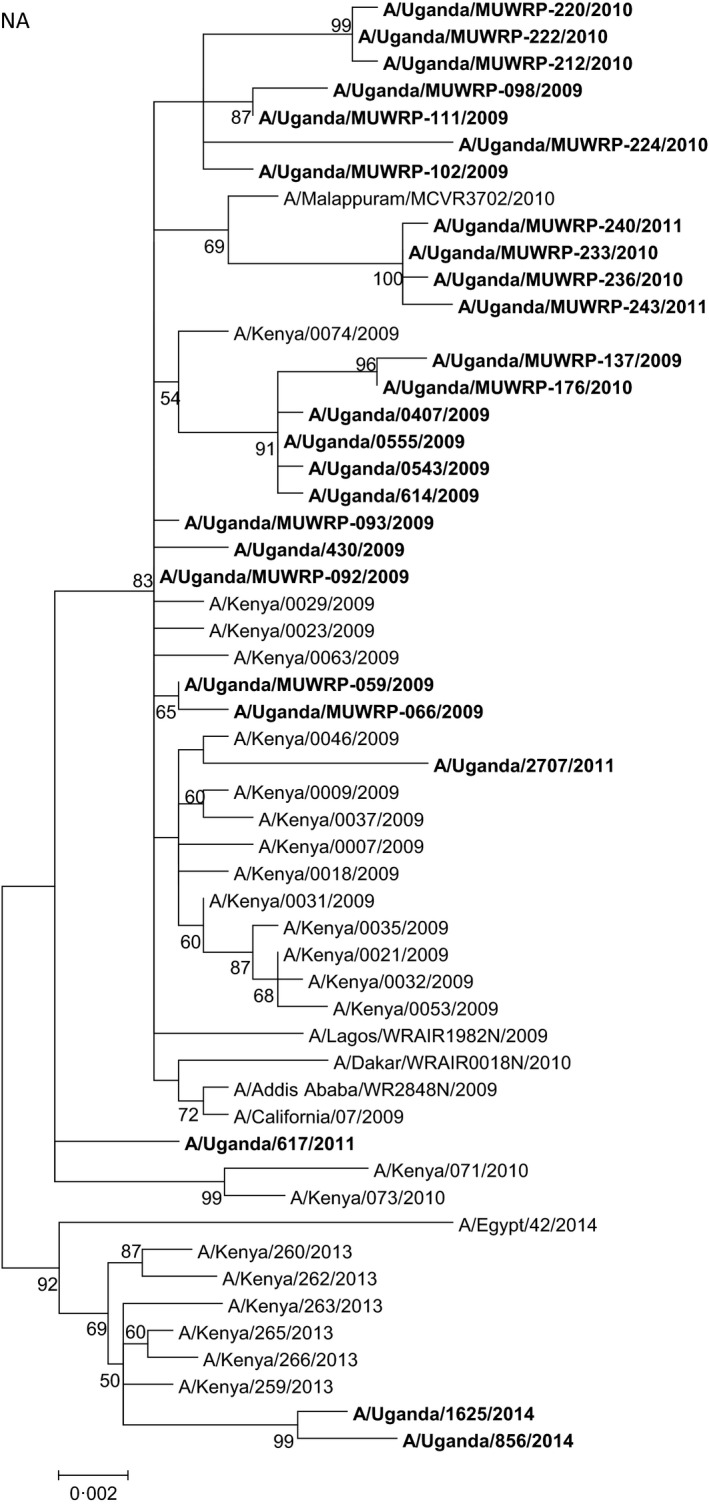
Phylogenetic tree of the neuraminidase (NA) gene segment of the Ugandan influenza A(H1N1)pdm09 viruses (in bold font) at the nucleotide level. The NA sequences of Ugandan H1N1 isolates were compared with relevant virus sequences available in GenBank: sequences from representative influenza A(H1N1)pdm09 isolates from Africa and closest blast hits from different regions of the world. Ugandan sequences available in the GISAID Epiflu database were also used (GISAID isolates ids: EPI_ISL_94767, EPI_ISL_105881, EPI_ISL_176803, EPI_ISL_176806, EPI_ISL_62338, EPI_ISL_65033, EPI_ISL_65034, EPI_ISL_68760, and EPI_ISL_71429). Sequences from vaccine strains [A/California/07/2009 (H1N1pdm09) and A/Brisbane/59/2007 (seasonal H1N1)] were also included. Bootstrap values (500 replicates) >50 are indicated on the nodes. *indicates partial sequence data

Phylogenetic analysis of NA also revealed a low genetic variation (genetic distance 0.1%–1.6%) at the nucleotide level (Fig. 3), but the strains isolated in different years could also be distinguished by their amino acid substitutions. Besides the common amino acid differences mentioned above, strains isolated from early 2009 were defined by the amino acid substitution N309D, and two isolates also had the amino acid substitutions M15V, I17V, and I188T, whereas strains isolated from late 2010 and 2011 were defined by amino acid substitutions R220K, Q313R, I389K, and V394I. One strain isolated from 2010 had a unique NA genotype, with amino acid substitutions Q39R, S70N, N190S, Q309S, and N386S.

**Figure 4:**
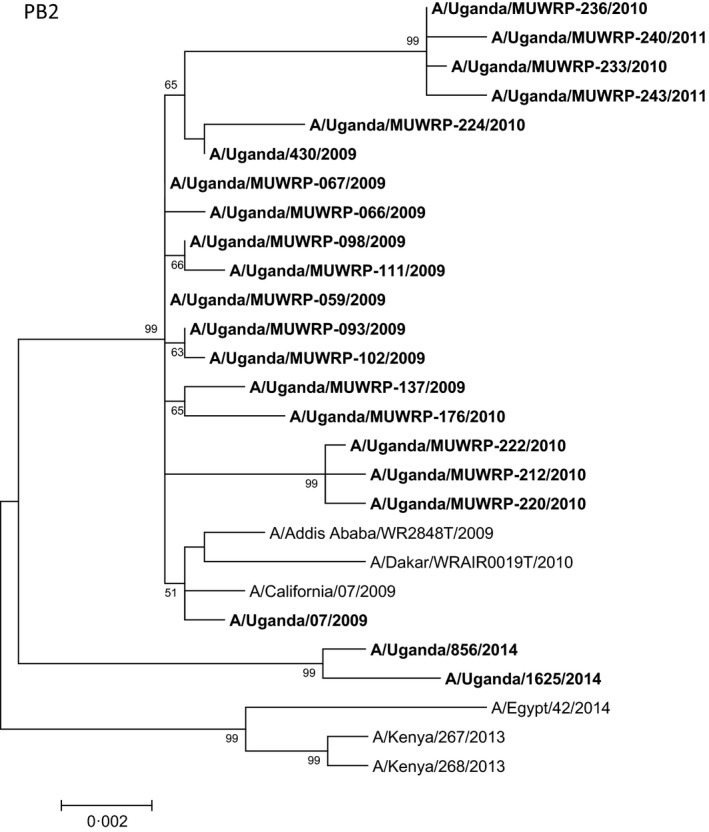
Phylogenetic tree of the polymerase 2 (PB2) gene segment of Ugandan influenza A(H1N1)pdm09 viruses (in bold font) at the nucleotide level. The PB2 sequences of Ugandan H1N1 isolates were compared with relevant virus sequences available in GenBank: sequences from representative influenza A(H1N1)pdm09 isolates from Africa and closest blast hits from different regions of the world. Ugandan sequences available in the GISAID Epiflu database were also used (GISAID isolates ids: EPI_ISL_176803, EPI_ISL_176806, EPI_ISL_62338, EPI_ISL_35548). Sequences from vaccine strains [A/California/07/2009 (H1N1)pdm09) and A/Brisbane/59/2007 (seasonal H1N1)] were also included. Bootstrap values (500 replicates) >50 are indicated on the nodes. *indicates partial sequence data

The phylogenetic tree of PB2 (Fig. 4) and other gene segments (data not shown) of the influenza A(H1N1)pdm09 strains exhibited a similar pattern of clustering of isolates. SF variant type analysis did not predict significant variations associated with increased virus fitness or transmission efficiency or significant antigenic differences. No molecular determinant of oseltamivir resistance was identified, but the determinant of amantadine resistance was suspected because of the Ser31Asn substitution in the M2 gene.

## 5 Discussion

Genome analysis helps understand the molecular evolution of viruses and other genetic factors unique to isolates that might help inform the formulation of vaccines for specific regions. While the influenza A(H1N1)pdm09 strains isolated in this study had specific amino acids that defined them, they were generally similar to the reference strains, with no significant antigenic variation from the vaccine strain A/California/07/2009.^8^ Consistent with findings from previous reports,^9^, ^10^, ^11^, ^12^ the isolates from our study showed low genetic diversity. Despite this, the viruses isolated in different seasons clustered closely in the phylogenetic trees and could fairly be separated from each other. The influenza A(H1N1)pdm09 strains completely replaced the seasonal H1N1 strains shortly after the 2009 pandemic, and these strains have not been isolated since that time, a phenomenon reported in other regions of the world.^13^

The limited information on whole‐genome sequences from influenza virus strains from sub‐Saharan Africa has constrained the region's contribution toward generating the information needed to develop new vaccines, therapies, and diagnostics for influenza. There are reported mutations in the influenza A(H1N1)pdm09 strains that can lead to increased fitness.^14^ However, on the basis of SF analysis, the isolates in our study did not carry amino acid substitutions that are associated with efficient transmission or fitness, although biological experiments would be needed to further confirm this observation. Genetic changes in influenza viruses often arise as a result of several years of re‐assortment among different subtypes of viruses.^15^ Most of the isolates in our study shared high similarity with contemporary reference strains from other regions, which indicates that the Uganda viruses could be because of the introduction of new viruses into Uganda from the rest of the world rather than independent evolution of variants within Uganda.

## 6 Conclusion

The sequence feature (SF) variant type analysis of the genomes of our isolates did not predict key mutations associated with functional, epitope, and structural features that are related to increased fitness and antigenic variations. Nevertheless, accumulated amino acid substitutions were identified and with the ability of these viruses to undergo rapid mutations, it is critical to maintain continuous surveillance to identify any important changes that might be associated with increase viral fitness and transmissibility.

## Disclaimer

The views expressed herein are those of the authors and do not represent those of the US Army Medical Research Directorate – Kenya, the Walter Reed Army Institute of Research, the US Army Medical Command, the US Department of the Army, or the US Department of Defense.

## References

[1] Sanicas M , Forleo E , Pozzi G , Diop D . A review of the surveillance systems of influenza in selected countries in the tropical region. Pan Afr Med J. 2014;19:121. 25745529 10.11604/pamj.2014.19.121.4280 PMC4341259

[2] Belanov SS , Bychkov D , Benner C , et al. Genome‐Wide Analysis of Evolutionary Markers of Human Influenza A(H1N1)pdm09 and A(H3N2) Viruses May Guide Selection of Vaccine Strain Candidates. Genome Biol Evol. 2015;27:7. 10.1093/gbe/evv240 PMC4700966 26615216

[3] CDC . Protocol of real time RTPCR for influenza A(H1N1). The WHO Collaborating Centre for influenza at CDC Atlanta: USA; 2009.

[4] Palmer DF , Dowdle WR , Coleman MT , Schild GC . Advanced laboratory techniques for influenza diagnosis. Immunology Series No. 6. Atlanta, United States Department of Health, Education and Welfare, 1975.

[5] Hoffman E , Stech J , Guan Y , Webster RG , Perez DR . Universal primer set for the full‐length amplification of all influenza A viruses. Arch Virol. 2001;146:2275–2289. 11811679 10.1007/s007050170002

[6] Hall TA . BioEdit: a user‐friendly biological sequence alignment editor and analysis program for Windows 95/98/NT. Nucleic Acids Symp Ser. 1999;41:4.

[7] Noronha JM , Liu M , Squires RB , et al. Influenza virus sequence feature variant type analysis: evidence of a role for NS1 in influenza virus host range restriction. J Virol. 2012;86:5857–5866. 22398283 10.1128/JVI.06901-11 PMC3347290

[8] Garten RJ , Davis CT , Russell CA , et al. Antigenic and genetic characteristics of swine‐origin 2009 A(H1N1) influenza viruses circulating in humans. Science. 2009;325:197–201. 19465683 10.1126/science.1176225 PMC3250984

[9] Baillie GJ , Galiano M , Agapow PM , et al. Evolutionary dynamics of local pandemic H1N1/2009 influenza virus lineages revealed by whole‐genome analysis. J Virol. 2012;86:11–18. 22013031 10.1128/JVI.05347-11 PMC3255882

[10] Galiano M , Agapow PM , Thompson C , et al. Evolutionary pathways of the pandemic influenza A (H1N1) 2009 in the UK. PLoS One. 2011;6:e23779. 21887318 10.1371/journal.pone.0023779 PMC3161082

[11] Sant'Anna FH , Borges LG , Fallavena PR , et al. Genomic analysis of pandemic and post‐pandemic influenza A pH1N1 viruses isolated in Rio Grande do Sul, Brazil. Arch Virol. 2014;159:621–630. 24114147 10.1007/s00705-013-1855-8

[12] de Vries RP , de Vries E , Martínez‐Romero C , et al. Evolution of the hemagglutinin protein of the new pandemic H1N1 influenza virus: maintaining optimal receptor binding by compensatory substitutions. J Virol. 2013;87:13868–13877. 24109242 10.1128/JVI.01955-13 PMC3838262

[13] Barr IG , Cui L , Komadina N , et al. A new pandemic influenza A(H1N1) genetic variant predominated in the winter 2010 influenza season in Australia, New Zealand and Singapore. Euro Surveill. 2010; 15. pii: 19692 10.2807/ese.15.42.19692-en 21034722

[14] Ye J , Sorrell EM , Cai Y , et al. Variations in the hemagglutinin of the 2009 H1N1 pandemic virus: Potential for strains with altered virulence phenotype? PLoS Pathog. 2010;6:e1001145. 20976194 10.1371/journal.ppat.1001145 PMC2954835

[15] Bush RM , Bender CA , Subbarao K , Cox NJ , Fitch WM . Predicting the evolution of human influenza A. Science. 1999;286:1921–1925. 10583948 10.1126/science.286.5446.1921

